# Renaming the SARS‐CoV‐2 Omicron sublineages as SARS‐CoV‐3 is contrary to nomenclature standards based on evolutionary and serological evidence

**DOI:** 10.1002/ctm2.924

**Published:** 2022-06-13

**Authors:** Zhongtian Qi, Jianbo Wu, Shibo Jiang

**Affiliations:** ^1^ Department of Microbiology Faculty of Naval Medicine Naval Medical University Shanghai China; ^2^ Key Laboratory of Medical Molecular Virology (MOE/NHC) Institute of Infectious Disease and Biosecurity School of Basic Medical Sciences Fudan University Shanghai China

1

A news article entitled ‘New versions of Omicron are masters of immune evasion’ authored by Gretchen Vogel was published in *Science* on 10 May 2022. She mentioned all three new sublineages of severe acute respiratory syndrome coronavirus 2 (SARS‐CoV‐2) Omicron variant, including BA.4 and BA.5 first detected in South Africa and BA.2.12.1 now spreading in the United States, as having a key escape mutation in L452, potentially explaining the reduced neutralising capacity in sera from vaccinated or BA.1‐infected‐but‐recovered individuals.^1^ The author further noted that Linfa Wang, Professor and Director at Duke‐NUS Medical School in Singapore, proposed renaming the new Omicron sublineages as SARS‐CoV‐3 based on their broad and dramatic immune escape profile.^1^ Not surprisingly, this idea has provoked wide discussion within the scientific community.

Some scientists have agreed with this proposal, but we and others do not, based on the following reasoning. Evolutionarily,^2^ SARS‐CoV‐2 wildtype shares approximately 79% and 55% sequence similarity with SARS‐CoV and Middle East respiratory syndrome coronavirus (MERS‐CoV), respectively, while its sequence similarities with Omicron BA.2, BA.2.12.1, BA.4 and BA.5 sublineages are 99.67%, 99.65%, 99.62% and 99.66%, respectively (Figure 1). Therefore, the proposed renaming is contrary to the principles of nomenclature, as suggested by the International Committee on Taxonomy of Viruses (ICTV), based on such consistently high sequence similarity between the new Omicron sublineages and SARS‐CoV‐2 wildtype.^3^, ^4^ Also, from the perspective of serotype, the distinct levels of antibody recognition induced by escape mutation among different sublineages of the same type of virus is not sufficient and reasonable for defining a novel virus. For example, the four serotypes of dengue virus (DENV), designated as DENV‐1, ‐2, ‐3 and ‐4, all belong to the same type of virus in the family Flaviviridae, genus *Flavivirus*, but showed limited levels of antibody cross‐protection among each other.^5^

**FIGURE 1:**
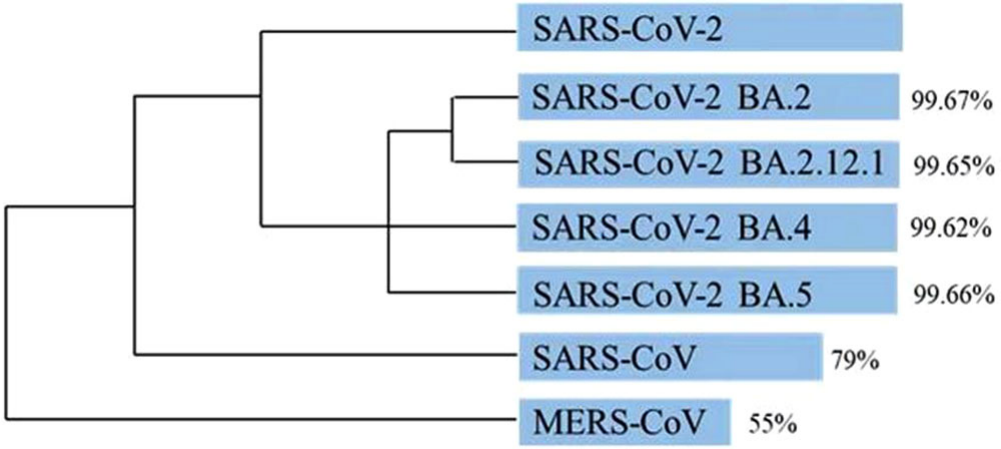
Phylogeny of coronaviruses related to SARS‐CoV‐2. The length of the blue bars represents the identity of the gene sequences in these viruses

During the recent wave of SARS‐CoV‐2 infection in Shanghai, a total of 620 911 cases were identified, including 57 260 symptomatic cases and 576 fatalities, until 16 May 2022. The vaccination rate among death cases was extremely low, only 4.97%.^6^ This number implies that the current vaccines used in China, including inactivated vaccines, adenovirus‐vector‐based vaccines and receptor‐binding domain (RBD)‐based subunit vaccines, as well as the mRNA‐based BNT162b2 vaccines, could still provide efficient protection against severe disease caused by different sublineages of the Omicron variant.^7^ Moreover, Shanghai has persisted in aiming for dynamic zero coronavirus disease 2019 (COVID‐19) at the community level. This highlights the importance of developing new vaccines targeting the emerging Omicron sublineages, as well as efficient therapeutic drugs, such as broadly neutralising antibodies specific for RBD and spike (S) protein and small‐molecule antiviral drugs targeting either RNA‐dependent RNA polymerase or SARS‐CoV‐2 proteases.

Adding together the reasons suggested above, it seems premature to propose a new name for several new Omicron sublineages. For a public already bewildered by the list of COVID‐19 variants of concern (VOCs), this action might cause further panic and misinformation campaigns when this so‐called ‘new species, SARS‐CoV‐3’, is nothing more than sublineages of SARS‐CoV‐2 Omicron variant.

## CONFLICT OF INTEREST

The authors declare that there is no conflict of interest that could be perceived as prejudicing the impartiality of the research reported.
